# Supplementary material for: Breast self-examination practice and its determinants among women in Ethiopia: A systematic review and meta-analysis
Source: PLoS One. 2021 Jan 14;16(1):e0245252. doi: 10.1371/journal.pone.0245252 (PMC7808636; doi:10.1371/journal.pone.0245252)
Supplement: S1 File — (DOC) [file pone.0245252.s002.doc]

# **Supplementary file 1: search strategy used for the systematic and Meta analysis on Breast self-examination practice and its determinants among women in Ethiopia: a systematic review and meta-analysis**

(((("early detection of breast cancer"[All Fields] OR ("breast self-examination"[MeSH Terms] OR ("breast"[All Fields] AND "self-examination"[All Fields]) OR "breast self-examination"[All Fields] OR ("breast"[All Fields] AND "self"[All Fields] AND "examination"[All Fields]) OR "breast self-examination"[All Fields])) OR (("breast neoplasms"[MeSH Terms] OR ("breast"[All Fields] AND "neoplasms"[All Fields]) OR "breast neoplasms"[All Fields] OR ("breast"[All Fields] AND "cancer"[All Fields]) OR "breast cancer"[All Fields]) AND ("diagnosis"[Subheading] OR "diagnosis"[All Fields] OR "screening"[All Fields] OR "mass screening"[MeSH Terms] OR ("mass"[All Fields] AND "screening"[All Fields]) OR "mass screening"[All Fields] OR "screening"[All Fields] OR "early detection of cancer"[MeSH Terms] OR ("early"[All Fields] AND "detection"[All Fields] AND "cancer"[All Fields]) OR "early detection of cancer"[All Fields]))) AND "practice"[All Fields]) AND (("female"[MeSH Terms] OR "female"[All Fields]) OR ("women"[MeSH Terms] OR "women"[All Fields]))) AND ("ethiopia"[MeSH Terms] OR "ethiopia"[All Fields]).
