# Supplementary material for: Breast self-examination practice and its determinants among women in Ethiopia: A systematic review and meta-analysis
Source: PLoS One. 2021 Jan 14;16(1):e0245252. doi: 10.1371/journal.pone.0245252 (PMC7808636; doi:10.1371/journal.pone.0245252)
Supplement: Attachment — Submitted filename: Response to Reviewers.docx [file pone.0245252.s004.docx]

| **Responses to editor’s comment**  **Editor’s comments, suggestions and question are considered and carefully revised the manuscript as per the suggestions and comments. I am very thankful to the potential editor for suggestions and comments, which substantially improved the manuscript** | |
| --- | --- |
| 1. Please ensure that your manuscript meets PLOS ONE's style requirements, including those for file naming. The PLOS ONE style templates can be found at  https://journals.plos.org/plosone/s/file?id=wjVg/PLOSOne_formatting_sample_main_body.pdf and  https://journals.plos.org/plosone/s/file?id=ba62/PLOSOne_formatting_sample_title_authors_affiliations.pdf | Thank you, dear editor, we have re-written the manuscript based on PLOS ONE's style requirements |
| Please attach a Supplemental file of the results of the individual components of the quality assessment, not just the overall score, for each study included. Please also explain the reasons, and number of studies excluded for each reason, in the flow diagram. Thank you. | Thank you, dear editor, we have attached supplementary file of the results of the individual components of the quality assessment in the current version of the revised manuscript (attached as **supplementary file 3, Microsoft excel format**) |
| Thank you for including the statement that "The primary online search for review was conducted between February 16 to May 21 2020.” Please revise this statement to clarify whether all databases were searched from inception, or if there were any limits placed on the publication dates in your search. | Thank you dear editor for the meticulous observation, we have included studies irrespective of their publication date through May 21 2020 (mentioned in the manuscript in method section, under sub heading Data extraction and quality assessment, **Line 163, Page 8)** |
| Please include captions for your Supporting Information files at the end of your manuscript, and update any in-text citations to match accordingly. Please see our Supporting Information guidelines for more  information: http://journals.plos.org/plosone/s/supporting-information. | We have attached the following supporting information at end of the manuscript,   - **PRISMA checklist**: Reporting Items for Systematic Review and Meta-Analysis (PRISMA-P) 2009 statement guideline - **Supplementary file 2**: search strategy used for the systematic and Meta-analysis on Breast self-examination practice and its determinants among women in Ethiopia: a systematic review and meta-analysis - **Supplementary file 3:** JBI Critical Appraisal Checklist for Studies Reporting Prevalence Data |
| We note that this manuscript is a systematic review or meta-analysis; our author guidelines therefore require that you use PRISMA guidance to help improve reporting quality of this type of study. Please upload copies of the completed PRISMA checklist as Supporting Information with a file name “PRISMA checklist” | We have uploaded copies of the completed PRISMA checklist as Supporting Information with a file name **“PRISMA checklist** |
| **Responses to the Reviewers' comment and question**  **Reviewer’s comments are considered and carefully revised the manuscript as per the suggestions and comments. I am very thankful to the potential reviewers for suggestions and comments, which substantially improved the manuscript** | |
| **Reviewer 1 comment and question** | **Responses Reviewer 1** |
| Data analyzed: Table 2 shows some characteristics of the studies included. Some include subjects to age 80, and others “older” subjects of unspecified age. These studies do not contribute to compliance with the WHO recommendation directed at women of reproductive age. If the results of these studies cannot be examined in relation to age I would suggest omitting them, or at least examining the effect of omitting them on the overall conclusion. | 1. We have included the age of the respondents under the Result section , sub heading Study identification, table 1 **(Page 11-13)** 2. **Dear reviewer based on the references we found and our best understanding, women above reproductive age group should be considered for breast self-examination especially in resource limited countries and most of the studies included under the current review included non-reproductive age group (**WHO, and other organizations recommended the starting age ( starting from age of 20 where the breast develops or matures) of breast self-examination, however the upper age wasn’t restricted or specified. A recommendation from WHO (1) shows that beginning at the age of 20, women should examine their breast every two or three years and, increasing one year from the age of 40. Studies shows that, the risk of breast cancer increases dramatically after menopause (2). In low resource counties including Ethiopia where mammography is not affordable, it is feasible to extend the breast self-examination practice beyond reproductive age.   ****Besides we tried to see the sub group analysis by categorizing the studies into reproductive age and non-reproductive age (** |
| The sampling methods used by the included studies are not clear. Did any use random sampling in a defined population? | We have assessed the sampling method as one of the criteria under the quality appraisal, in addition we have prepared a separate file named sampling method (from the total of 41 studies included, 39 (95% of them used random method, of which 10 (25.6% used systematic random sampling) |
| It would also be useful to indicate in Table 2 which of the regions of Ethiopia included are rural or urban. | Thank you so much dear reviewer, we have included one additional column for residence under result section, sub heading study identification **(page 11-13),** 31 (80.4% of the included studies were from urban areas, the rest 19.6 are from urban and rural regions. **Additionally we conducted sub group analysis to see the variation accounted due to residence** ( |
| There is no definition of compliance with BSE- can a distinction be made between never, occasional and regular use? Examples of the wording of questions used by studies re BSE would be useful. | Dear reviewer thank you so much, we have included the definition of the outcome variable, Examples of the wording of questions used by studies and we also included the independent variable list too under the method section (Line **150** **-160**), **Page 7 to 8** |
| Analysis of determinants of BSE: The analysis of factors associated with practice of BSE appears to be entirely univariable. Variables such as educational level, occupation, knowledge of breast cancer etc. may well be related. Multivariable analysis would identify which of the candidate variables are independently associated with BSE practice. | Dear reviewer we applied the univariable analysis based on our knowledge and understanding of the following concept: Price MJ et al, in Cochrane review stated that When review has several outcomes of interest , and the effect estimates for these outcomes may be correlated within primary studies, because the same patients provide data towards them. Standard univariate meta‐analysis (UVMA) methods do not account for this correlation. In recognition of this, multivariate meta‐analysis (MVMA) models have been developed. In addition it was emphasized that multivariate meta‐analysis is suggested especially where there are missing data for some outcomes or where post estimation modelling requires intervention effect estimates for multiple correlated outcomes (3). Which we believe do not apply to our current study. |
| The discussion does not include any account of variation in opinions and data concerned with the usefulness of BSE and the effects of BSE on mortality from breast cancer, although most of this literature comes from Europe of North America. | Dear reviewer, thank you for pointing out this very important point which we failed to address, we have included the points under discussion **(Line 351-365)** |
| **Reviewer 2 comment and question** | **Responses to Reviewer 2** |
| Articles were often not used, as in the Abstract, where it starts "Survival rate from ..." when it should say "The survival rate...." | Corrected as per the suggestion (**line 26** of the abstract **and 91** in introduction section ) |
| The number of subjects should be given in the Abstract as well as the number of articles included. Also, it gives the lowest percentage of breast self-examination as being "...among the general population".; What does this mean, an average of the country? In the conclusion, it is stated "Understanding the syndemic factors..." I am not sure "syndemic" is the proper word to use here. | - Thank you dear reviewer, we have included the number of subjects and number of articles in the Abstract section under result subheading, (**Line 41**, **Page 2)** - Dear reviewer, in the current review we have four different population groups (health professional, students, teachers and the general population in the community), during the subgroup analysis the highest prevalence of breast self-examination was among health professionals and the lowest prevalence was among the general population in the current review) - The We apologize for the improper word use, the word syndemic, it rephrased in the paragraph **(Line 54 -55, Page 3** |
| In the Introduction, it says that breast self-examination has been promoted as a screening method. In the fourth paragraph, line 83, it states that "BSA is recommended for raising awareness ....rather than a screening method." BSA stands for breast self-awareness; do you actually mean BSE (breast self-exam)? | Dear reviewer thank you so much for pointing that out, we have comprehended the paragraph in incorrect way, we apologize for that, and yes it was supposed to be BSE. We have corrected based on your corrections and modified the paragraph too (**Line 88-90)** |
| In the next paragraph, it states "The practice of breast self-examination varies across Ethiopia with different variations across women population." Do you mean across the population of women? | Thank you so much for your directions, it is corrected as across the population of women **(line 95)** |
| In Table 1, Study Populations of Students, Employees, and Teachers are given with no age ranges or other details. Have these been omitted, or did the studies used not have this information? Below Table 1 in Prevalence of Breast self-examination practices in Ethiopia, a P value of 0.000 is given. Is this possible, or was a number left out? | 1. We have added one additional column and included the age of respondents in table one **(Page 11-13)** 2. Dear reviewer we have corrected it as p<0.001under result section, sub heading prevalence of breast self-examination **practice in Ethiopia, line 252, page 14** (The level of statistical significance is expressed as a *p*-value between 0 and 1. Some statistical software like STAT sometimes gives *p* value .000 which is impossible and must be taken as *p*< .001) |
| In the Discussion section, possibly paragraph 3, lines 359 and 360, "...calls for population based breast self-examination nationwide awareness creation programs...." Do you mean "...show a need for the creation of nationwide awareness and population based breast self-examination programs"? Two paragraphs below, line 371, it says "... (health care workers where the study participants)" Do you mean health care workers were the participants? | 1. Thank you for pointing out the idea, the paragraph is modified according to your suggestion as: shows a need for the creation of nationwide awareness and population based breast self-examination programs, **we have also added literatures to support and strengthen the idea.** (**Line** 399-404, Page 2. Thank you very much for your meticulous observation to the errors we were unable to detect, corrected as per your observation as: yes health care workers were the participants **(line 415)** |
| **Additional corrections made by the authors** | |
| Authors' Contributions corrected as PLOS ONE requirement | **Line 503-517** |
| Acknowledgment section was mistakenly repeated and, therefore, deleted from the manuscript. | Deleted from **line 496** |
| On discussion section, additional emphasis has been made on the contribution of educational programs on breast self-examination practice. | **Line 396-401, Page 24** |
| Subgroup analysis done accounting for variations in residence and age category and supported with discussion | **Result section**, subheading, Prevalence of Breast self-examination practice in Ethiopia, **Table 2**, **Page 17**  **Discussion, Line 443-555, Page 27-28** |

**Reference**

1.	World Health Organization. Guidelines for the early detection and screening of breast cancer. Geneva, switzerland; 2006.

2.	McCready T, Littlewood D, Jenkinson J. Breast self-examination and breast awareness: a literature review. 2005;14(5):570-8.

3.	Price MJ, Blake HA, Kenyon S, White IR, Jackson D, Kirkham JJ, et al. Empirical comparison of univariate and multivariate meta-analyses in Cochrane Pregnancy and Childbirth reviews with multiple binary outcomes. Research synthesis methods. 2019;10(3):440-51.
