# Supplementary material for: Breast self-examination practice and its determinants among women in Ethiopia: A systematic review and meta-analysis
Source: PLoS One. 2021 Jan 14;16(1):e0245252. doi: 10.1371/journal.pone.0245252 (PMC7808636; doi:10.1371/journal.pone.0245252)
Supplement: Attachment — Submitted filename: Response to Reviewers.docx [file pone.0245252.s005.docx]

| **Responses to editor’s comment**  **Editor’s suggestions are considered and carefully revised the manuscript. I am very thankful to the potential editor for suggestions and comments, which substantially improved the manuscript** | |
| --- | --- |
|  |  |
| a) If there are ethical or legal restrictions on sharing a de-identified data set, please explain them in detail (e.g., data contain potentially identifying or sensitive patient information, data are owned by a third-party organization, etc.) and who has imposed them (e.g., a Research Ethics Committee or Institutional Review Board, etc.). | Dear editor, there are no ethical or legal restrictions on sharing a de-identified data set, please explain them in detail (e.g., data contain potentially identifying or sensitive patient information, data are owned by a third-party organization. |
| b) Note that it is not acceptable for an author to be the sole named individual responsible for ensuring data access. Please provide contact information (preferably email) for a data access committee, ethics committee, or another institutional body to which data requests may be sent. | Dear editor, We have updated the data availability statement in the and included all the data’s which are related to the current manuscript as “supplementary files” |
| c) If there are no restrictions, please upload the minimal anonymized data set necessary to replicate your study findings to a stable, public repository and provide us with the relevant URLs, DOIs, or accession numbers. Please see <http://www.bmj.com/content/340/bmj.c181.long> for guidelines on how to de-identify and prepare clinical data for publication. For a list of recommended repositories, please see <http://journals.plos.org/plosone/s/data-availability#loc-recommended-repositories.> You also have the option of uploading the data as Supporting Information files, but we would recommend depositing data directly to a data repository if possible. | Dear editor, We have uploaded the data Supporting Information files |
